# A MIMO-SAR Tomography Algorithm Based on Fully-Polarimetric Data

**DOI:** 10.3390/s19224839

**Published:** 2019-11-06

**Authors:** Lingyu Kong, Xiaojian Xu

**Affiliations:** School of Electronics and Information Engineering, Beihang University, Beijing 100191, China; konglingyu@buaa.edu.cn

**Keywords:** polarimetric, SAR tomography, MIMO radar

## Abstract

A fully-polarimetric unitary multiple signal classification (UMUSIC) tomography algorithm is proposed, which can be used for acquiring high-resolution three-dimensional (3D) imagery, in a polarimetric multiple-input multiple-output synthetic aperture radar (MIMO-SAR) with a small number of baselines. In terms of the elevation resolution, UMUSIC provides an improvement over standard MUSIC by utilizing the conjugate of the complex sample data and converting the complex covariance matrix into a real matrix. The combination of UMUSIC and fully-polarimetric data permits a further reduction of the noise of the sample covariance matrix, which is obtained through pixel averaging of multiple two-dimensional (2D) images. Considering the consistency of four polarizations, this algorithm not only makes scattering centers have the same estimated height in four polarizations, but it also improves the estimation accuracy. Simulation results show that this algorithm outperforms the popular distributed compressed sensing (DCS). Image processing of measured data of an aircraft model using a multiple-input multiple-output synthetic aperture radar (MIMO-SAR) with six baselines is presented to validate the proposed algorithm.

## 1. Introduction

Multiple-input multiple-output synthetic aperture radar (MIMO-SAR) is an enabling technique capable of imaging a target [1,2,3,4,5,6,7], which is different from a rail synthetic aperture radar (SAR) and a turntable inverse synthetic aperture radar (ISAR). Two-dimensional (2D) virtual apertures can be synthesized through different combinations of transceiver antenna elements. A large number of virtual apertures in the cross-range and elevation directions are beneficial to obtain high-resolution three-dimensional (3D) radar images [4,5,6], but they also result in a high cost and large size of radar systems due to the increase of the number of antenna elements. When the measured target has several scattering centers in an elevation direction, such as airplanes, an affordable array strategy can be adopted: the priority is given to ensuring adequate cross-range virtual apertures for high-resolution two-dimensional (2D) radar images [7], and then a small number of elevation virtual apertures ensure high-resolution 3D radar images by SAR tomography [8,9,10,11,12,13,14,15,16,17,18].

The reconstruction quality of SAR tomography depends on the product of the number of baselines and the signal-to-noise ratio (SNR) [8]. When the number of baselines is limited, the SNR of radar images can be equivalently improved by filtering [8,9], auxiliary information [10,11,12], and polarization [13,14,15,16,17]. The SNR can be improved by integrating nonlocal filtering into the compressed sensing (CS) algorithm and a reasonable reconstruction of buildings from only seven baselines is feasible [8]. In addition, [9] investigates the possibility related to the use of a multi-looking approach for fine resolution analysis of ground structures that combines SAR tomography. Filtering consisting of averaging pixels is bound to reduce the range-azimuth resolution and therefore is not suitable for high-resolution radar images of artificial targets. The auxiliary information added to the standard Capon and multiple signal classification (MUSIC) algorithms can be exploited to reduce the ambiguity and resolve the superimposition of the scatterers in the case of a limited number of radar images [10]. Atmospheric phases for SAR tomography in mountainous regions are regressed against the spatial coordinates in map geometry at persistent scatterers locations [11]. The high-resolution 3D positions of a large amount of natural scatterers are obtained by a geodetic SAR tomography framework that fuses SAR tomography and SAR image geodesy compensating SAR measurement error [12]. The auxiliary information can effectively improve the image quality, but it needs to be obtained by other technologies which increases the complexity of the algorithm and the cost of the imaging. A distributed compressed sensing (DCS) algorithm based on fully-polarimetric data is proposed in [13,14,15,16] to improve the accuracy of the estimation. However, the CS algorithm suffers from a high computational expense and is hard to extend to fast practice [18]. In [17], a comparison among tomograms obtained in different polarizations is made to analyze how polarimetry can enhance target signatures.

To address these problems, the combination of spectral analysis and full polarization is an attractive way to improve resolution and processing speed for a small number of baselines. This paper explores a fully-polarimetric unitary multiple signal classification (UMUSIC) technique for polarimetric MIMO-SAR tomography [7]. The remainder of the paper is organized as follows. Section 2 describes a signal model based on fully-polarimetric data. A fast and high-resolution UMUSIC algorithm is developed in Section 3. In Section 4, two algorithms are compared through simulation of different point scatterers. Finally, Section 5 contains measured tomography results of an aircraft model to validate the proposed algorithm.

## 2. Polarization Signal Model

**Figure 1:**
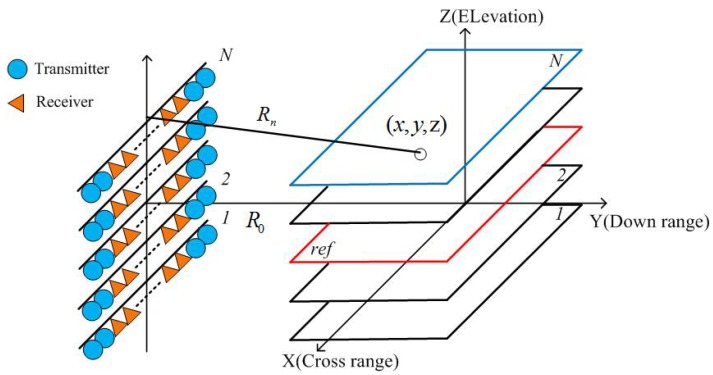
Geometry of multiple-input multiple-output synthetic aperture radar (MIMO-SAR) tomography.

SAR tomography allows us to obtain 3D imagery to describe the electromagnetic property of illuminated objects. The geometry of MIMO-SAR tomography is shown in Figure 1, where *x,y,z* denote coordinates originating from the center of the imagery scene, *R_n_* represents the distance from the target to the *n*th baseline, and *R*_0_ is the projection distance from the radar to the center of the imagery scene on the *y*-axis. The orange triangles and blue circles denote receivers and transmitters, respectively. Each baseline represents a linear array, where transmitters are at both ends of the array and receivers are in the middle of the array. The 2D image for the *n*th baseline is represented by the following form [19].

(1) $$g_{n}(x,y,z_{n})=\int_{-h/2}^{h/2}\sqrt{\overset{⌢}{\sigma }(x,y,z)}e^{-j\frac{4\pi }{\lambda }R_{n}}dz$$

where $\sqrt{\overset{⌢}{\sigma }(x,y,z)}$ denotes the target scattering function that needs to be solved, *h* is the height of the imagery scene, *λ* represents the wavelength and *z_n_* refers to the *z*-coordinates of the *n*th baseline. Under the Born weak-scattering approximation, $R_{n}$ representing the distance from the point target at (*x, y, z*) to the *n*th baseline, is approximated as:

(2) $$R_{n}=\sqrt{{\left(y+R_{0}\right)}^{2}+{\left(z-z_{n}\right)}^{2}}\approx y+R_{0}+\frac{z_{n}^{2}+z^{2}-2z_{n}z}{2R_{0}}$$

The first three terms in (2) are irrelevant to *z*, the fourth term is the residual phase term that can be merged into $\sqrt{\overset{⌢}{\sigma }(x,y,z)}$, and the fifth term is the phase term for imaging in the elevation direction. We can choose one of *N* baselines as a reference baseline as:

(3) $$g_{ref}(x,y,z_{ref}){=e}^{-j\frac{4\pi }{\lambda }(y+R_{0}+\frac{z_{n}^{2}}{2R_{0}})}$$

After phase compensation, the *N* 2D images $g_{n}^{\prime }(x,y,z_{n})$ and ${\overset{⌢}{\sigma }}^{\prime }(x,y,z)$ are in a Fourier transform relation.

(4) $$g_{n}^{\prime }(x,y,z_{n})=g_{n}(x,y,z_{n})g_{ref}^{*}(x,y,z_{ref})=\int_{-h/2}^{h/2}{\overset{⌢}{\sigma }}^{\prime }(x,y,z)e^{-j2\pi w_{n}z}dz$$

with

(5) $$w_{n}=\frac{2z_{n}}{\lambda R_{0}}$$

(6) $${\overset{⌢}{\sigma }}^{\prime }(x,y,z)=\sqrt{\overset{⌢}{\sigma }(x,y,z)}e^{-j\frac{2\pi z^{2}}{\lambda R_{0}}}$$

where ${\overset{⌢}{\sigma }}^{\prime }(x,y,z)$ integrated with the residual phase term still has the same amplitude as $\sqrt{\overset{⌢}{\sigma }(x,y,z)}$, which has no effect on the 3D imagery. $g_{n}^{\prime }(x,y,z_{n})$ can be discretized as the multiplication of two matrices.

(7) $$g_{n}^{\prime }(x,y,z_{n})=\sum_{l=1}^{L}{\overset{⌢}{\sigma }}^{\prime }(x,y,z_{l})e^{-j2\pi w_{n}z_{l}}=a_{n}s$$

with

(8) $$a_{n}=[e^{-j2\pi w_{n}z_{1}},e^{-j2\pi w_{n}z_{2}},\ldots ,e^{-j2\pi w_{n}z_{L}}]$$

(9) $$s={\left[{\overset{⌢}{\sigma }}^{\prime }(x,y,z_{1}),{\overset{⌢}{\sigma }}^{\prime }(x,y,z_{2}),\ldots ,{\overset{⌢}{\sigma }}^{\prime }(x,y,z_{L})\right]}^{T}$$

where *L* represents the number of scattering centers in the elevation direction. Before imaging, the imagery scene in the elevation direction needs to be divided into many discrete points to represent the range of *L*. As a consequence, the matrix $a_{n}$ is very large, which is the reason why the imaging algorithm takes a long time to locate scattering centers and determine *L* in the simulation and measurement. In combination with *N* 2D images, the polarimetric tomography model is given by:

(10) $$g=\left[\begin{array}{c} g_{1}^{\prime }(x,y,z_{1})\\ g_{2}^{\prime }(x,y,z_{2})\\ \vdots \\ g_{N}^{\prime }(x,y,z_{N}) \end{array}\right]=\left[\begin{array}{cccc} e^{-j2\pi w_{1}z_{1}} & e^{-j2\pi w_{1}z_{2}} & \cdots & e^{-j2\pi w_{1}z_{L}}\\ e^{-j2\pi w_{2}z_{1}} & e^{-j2\pi w_{2}z_{2}} & \cdots & e^{-j2\pi w_{2}z_{L}}\\ \vdots & \vdots & \ddots & \vdots \\ e^{-j2\pi w_{N}z_{1}} & e^{-j2\pi w_{N}z_{2}} & \cdots & e^{-j2\pi w_{N}z_{L}} \end{array}\right]s=As$$

(10) can be further developed by merging with the fully-polarimetric data.

(11) $$G=[\begin{array}{cccc} g_{HH} & g_{HV} & g_{VH} & g_{VV} \end{array}]={\left[a_{1},a_{2},\ldots ,a_{N}\right]}^{T}[\begin{array}{cccc} s_{HH} & s_{HV} & s_{VH} & s_{VV} \end{array}]=AS$$

where **G** denotes the *N×4* 2D imagery matrix, **S** refers to the *L×4* 3D imagery matrix, and **A** is the *N × L* transformation matrix.

## 3. Tomography Algorithm

Multiple signal classification (MUSIC) is a spectral analysis algorithm based on the eigen decomposition of the sample covariance matrix. It is necessary to reduce the matrix noise by some techniques, including snapshot in direction-of-arrival and multi-looking in SAR tomography that also leads to a decrease in range-azimuth resolution [13]. In order to dispel the influence, we employ fully-polarimetric data and their conjugation to obtain the matrix.

The polarization tomography model (11) including Gaussian white noise matrix W is rewritten as:

(12) G=AS+W

The covariance matrix R is given by:

(13) $$\begin{array}{l} R=E\{GG^{H}\}=E\{\left[AS+W\right]\left[S^{H}A^{H}+W^{H}\right]\}\\ =AE\{SS^{H}\}A^{H}+E\{WW^{H}\}=APA^{H}+\sigma^{2}I \end{array}$$

where $\sigma^{2}I=diag\left\{{\left|\sigma_{1}\right|}^{2}\cdots {\left|\sigma_{N}\right|}^{2}\right\}$. The Hermite matrix $APA^{H}$ composed of the positive definite diagonal matrix P and the column full rank matrix A can be eigen decomposed to obtain the noise subspace. 

Multi-sample data is critical for the MUSIC algorithm to ensure a high-quality covariance matrix. In SAR tomography, the average of pixels with the same scattering characteristics is called multi-looking. However, the number of these pixels is limited, and the average processing also reduces the range-azimuth resolution. The combination of observed data and their conjugation can be equivalent to doubling the number of these pixels, which not only improves the estimation accuracy but also solves the problem of coherent signal estimation. To force the Hermite property of $APA^{H}$, the average of the covariance matrices is computed from forward and backward data samples [20].

(14) $$R_{M}=\frac{1}{2}(R+J_{N}R^{*}J_{N})=A\tilde{P}A^{H}+\sigma^{2}I$$

with

(15) $$\tilde{P}=\frac{1}{2}(P+DP^{*}D^{H})$$

(16) $$D=diag\left\{e^{j2\pi (w_{N}+w_{1})z_{1}}\ldots e^{j2\pi (w_{N}+w_{1})z_{L}}\right\}$$

where superscript ^*^ represents the conjugation, $J_{N}$ denotes the *N*×*N* exchange matrix with ones on its antidiagonal and zeros elsewhere. In order to reduce the computational complexity, $R_{M}$ can be transformed into a real covariance matrix by unitary transformation.

(17) $$R_{U}=Q_{N}^{H}R_{M}Q_{N}^{}=Q_{N}^{H}A\tilde{P}A^{H}Q_{N}^{}+\sigma^{2}I$$

where $Q_{N}^{}$ is any *N*×*N* unitary matrix to satisfy column conjugate symmetry. A simple form can be chosen as [21]

(18) $$Q_{N:\mathrm{even}}=\frac{1}{\sqrt{2}}\left[\begin{array}{cc} I_{N/2} & jI_{N/2}\\ J_{N/2} & -jJ_{N/2} \end{array}\right]$$

(19) $$Q_{N:odd}=\frac{1}{\sqrt{2}}\left[\begin{array}{ccc} I_{(N-1)/2} & 0 & jI_{(N-1)/2}\\ 0 & \sqrt{2} & 0\\ J_{(N-1)/2} & 0 & -jJ_{(N-1)/2} \end{array}\right]$$

As mentioned in (14) and (17), the real covariance matrix $R_{U}$ can be rewritten as:

(20) RU=12(QNHRQN+QNHJNR*JNQN)=12(QNHRQN+(QNH)*R*JNQN*)=Re{QNHRQN}

The real matrix is eigen decomposed as:

(21) Re{QNHRQN}=∑i=1NλiuiuiH+σ2∑i=1NuiuiH=∑i=1LλiuiuiH+σ2∑i=1NuiuiH

where $\lambda_{1}$,…,$\lambda_{N}$ are eigenvalues and $u_{1}$,…,$u_{N}$ represent corresponding orthogonal normalized eigenvectors. Among *N* eigenvectors of the $R_{U}$, *L* eigenvalues are related to the signal, and *N-L* eigenvalues are related to the noise. By using the noise subspace $E_{N}=span\{u_{N-L},\ldots ,u_{N}\}$, the fully-polarimetric pseudo-spectrum is expressed as:

(22) $$P_{MUSIC}^{FP}(w)=\frac{1}{A^{H}(w)E_{N}E_{N}^{H}A(w)}$$

We can find out the peaks of the spectrums to locate different scattering centers in the elevation dimension and estimate the scattering intensity by the least square method (LSM) in four polarizations.

(23) $$S={\left(A^{H}A\right)}^{-1}A^{H}G$$

where A is updated according to positions of the scattering centers.

## 4. Simulation

**Figure 2:**
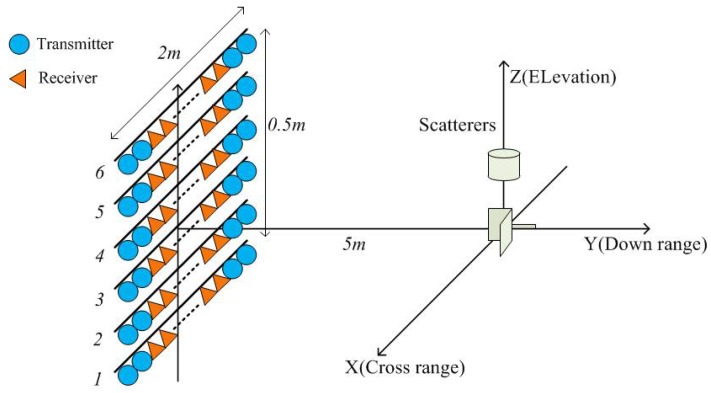
Simulation scene of the MIMO-SAR tomography.

**Figure 3:**
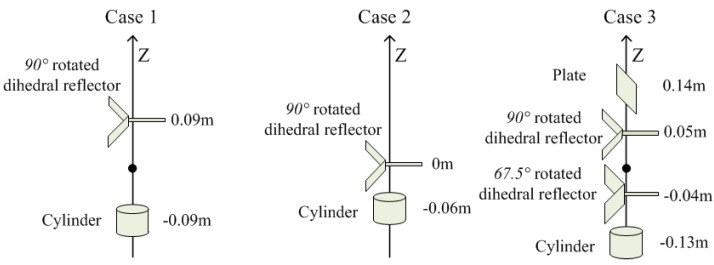
Positions of point scatterers in three cases.

We adopt the fully-polarimetric DCS as the comparison item. Considering crosstalk, noise, and dispersion, point scatterers with typical polarimetric scattering matrices (PSMs) are simulated to generate return signals. The simulation scene is shown in Figure 2, where scatterers with different heights are located in the coordinate origin. The working frequency is 8 GHz–12 GHz. The down-range, cross-range and elevation Rayleigh limits of the MIMO radar are 0.037 m, 0.047 m, and 0.188 m, respectively. As shown in Figure 3, we simulate three cases to compare the two algorithms.

### 4.1. Case 1: Two Point Scatterers with a Spacing of 0.18 m

**Figure 4:**
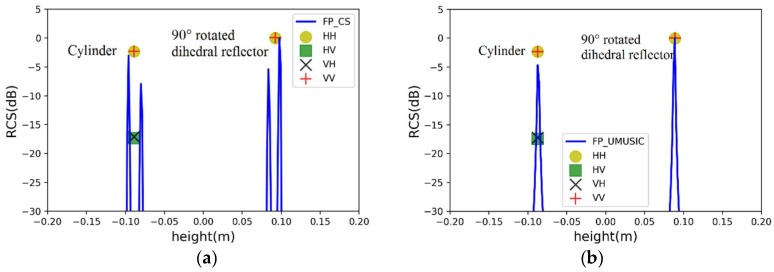
Estimated results of two point scatterers with a spacing of 0.18 m by (**a**) fully-polarimetric distributed compressed sensing (DCS) and (**b**) fully-polarimetric unitary multiple signal classification (UMUSIC).

A cylinder and a 90° rotated dihedral reflector are located at −0.09 m and 0.09 m in the elevation dimension, respectively. When the two scatterers spacing is 0.18 m (close to the Rayleigh limit), the simulation results of the two algorithms are seen in Figure 4, where lines represent pseudo-spectrums and points denote estimated results including height and scattering intensity of scatterers. 

Two fully-polarimetric algorithms make scatterers have the same estimated height in four polarizations. The specific estimated results are listed in Table 1. The PSMs of the two scatterers are estimated, where the scattering intensity of the cylinder return signal at −0.09 m is inconsistent with the truth because of polarimetric distortion. In Figure 4a, it is noteworthy that the pseudo-spectrum of CS is leaked to form false scattering points. There are two main reasons for signal leakage [13]: on the one hand, if the regularization parameter is too small in the optimization model, it can lead to over-fitting of data; on the other hand, the observed data does not satisfy the sparsity in the unit orthogonal basis. Therefore, it is necessary to use a sliding window to suppress signal leakage.

### 4.2. Case 2: Two Point Scatterers with a Spacing of 0.06 m

**Figure 5:**
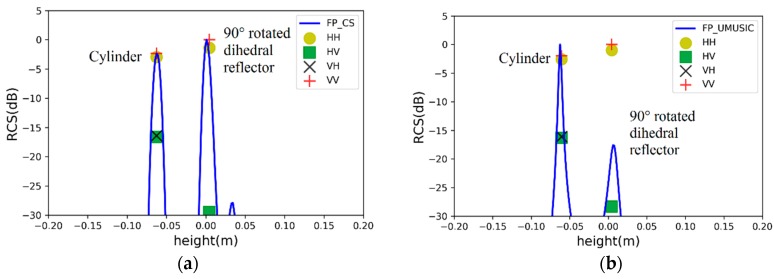
Estimated results of two point scatterers with a spacing of 0.06 m by (**a**) fully-polarimetric DCS and (**b**) fully-polarimetric UMUSIC.

When the spacing is reduced to 0.06 m (one-third of elevation Rayleigh limit), the estimated results of the two algorithms are displayed in Figure 5 and Table 2. Two fully-polarimetric algorithms still have high-resolution. Furthermore, polarimetric distortion of the cylinder return signals becomes more severe as the spacing decreases. Consequently, polarimetric calibration is necessary for a fully-polarimetric radar system.

### 4.3. Case 3: Four Point Scatterers with a Spacing of 0.09 m

**Figure 6:**
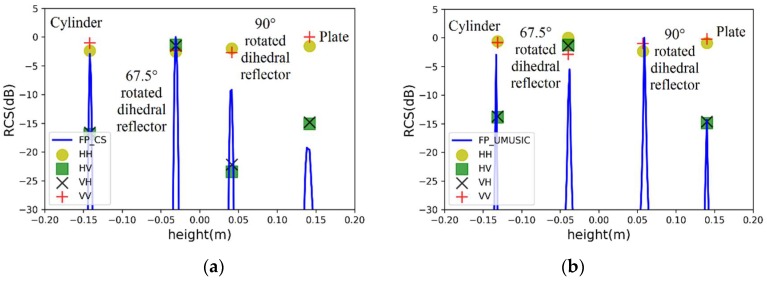
Estimated heights of four point scatterers with a spacing of 0.09 m by (**a**) fully-polarimetric DCS and (**b**) fully-polarimetric UMUSIC.

The four point scatterers are a cylinder, a 67.5° rotated dihedral reflector, a 90° rotated dihedral reflector, and a plate and their PSMs are listed in Table 3. According to the MIMO configuration, the bistatic angles of all transceiver channels are less than 10°. To simplify the simulation, we assume that the PSMs listed in Table 3 are applicable to all transceiver channels. It can be seen from Figure 6 that the pseudo-spectrums of two fully-polarimetric algorithms are not affected when the number of scatterers increase. We summarize the estimation results in Table 4, which demonstrates the estimation accuracy of fully-polarimetric UMUSIC is higher than that of the fully-polarimetric DCS. The CS, which is essentially an optimization problem, needs to be solved iteratively, therefore, its processing speed is bound to be limited by the number of iterations. The simulation results show that for a pixel, the processing speed of the fully-polarimetric UMUSIC is more than five times faster than that of the fully-polarimetric DCS in the same computing condition.

## 5. Experiment

**Figure 7:**
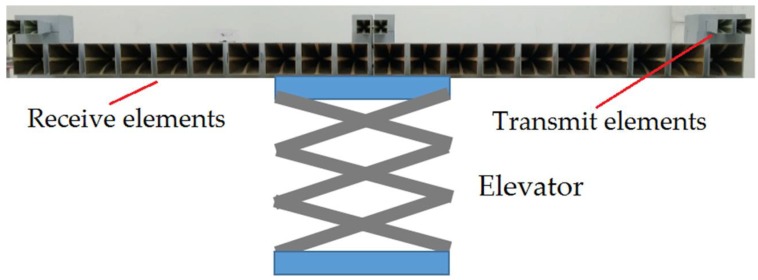
An experimental polarimetric MIMO array.

**Figure 8:**
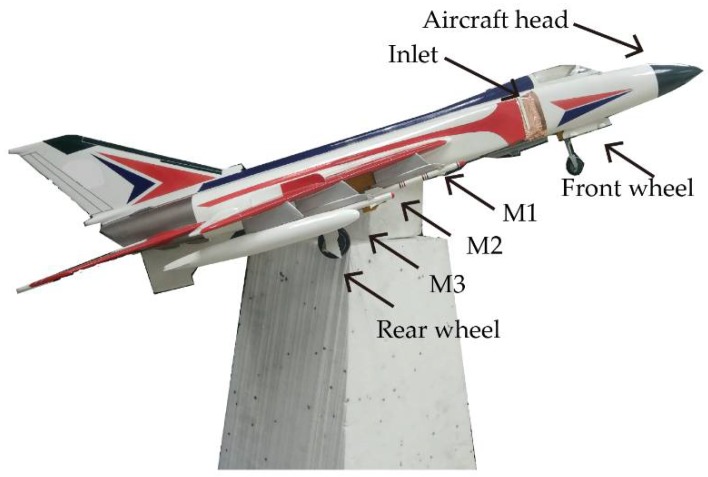
Aircraft model.

An experimental polarimetric MIMO array has been upgraded based on the radar system in [7], and baselines with different heights are controlled by an elevator. It can be seen from Figure 7 that the polarimetric MIMO array consists of 20 receive elements and 6 transmit elements, where the combinations among them synthesize 80 transceiver channels. The measured target is an aircraft model with an elevation angle of 16 degrees on a foam support, as shown in Figure 8. M1, M2, and M3 represent three missile models mounted on the wing, respectively. To avoid complex scattering properties of cavity structures, the inlet of the aircraft model is sealed with copper foils. The measurement parameters are the same as the simulation parameters in Section 4.

**Figure 9:**
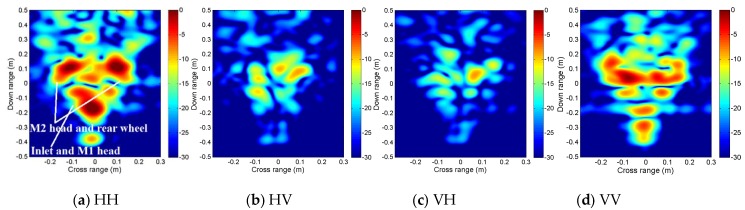
Four 2D images obtained from a single baseline: (**a**) HH, (**b**) HV, (**c**) VH, and (**d**) VV.

**Figure 10:**
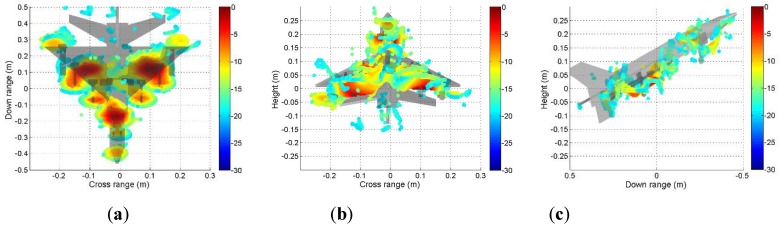
Tomography results in HH polarization. Three views of the airplane model are shown: (**a**) top view; (**b**) bottom view; (**c**) side view.

**Figure 11:**
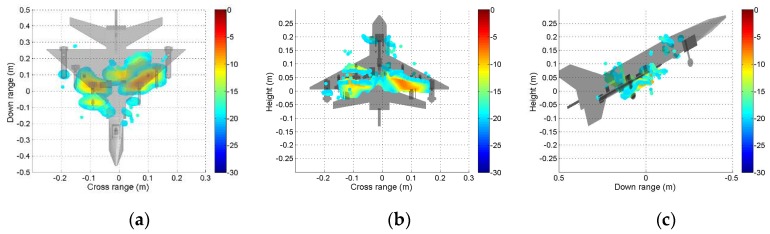
Tomography result in HV polarization. Three views of the airplane model are shown: (**a**) top view; (**b**) bottom view; (**c**) side view.

**Figure 12:**
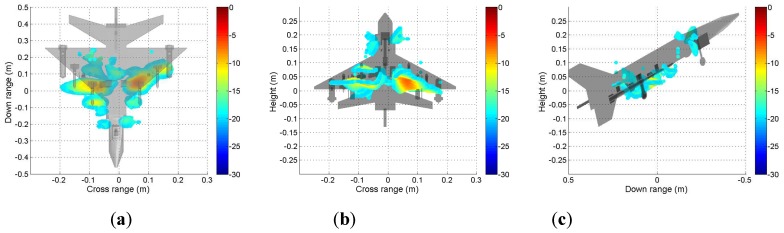
Tomography result in VH polarization. Three views of the airplane model are shown: (**a**) top view; (**b**) bottom view; (**c**) side view.

**Figure 13:**
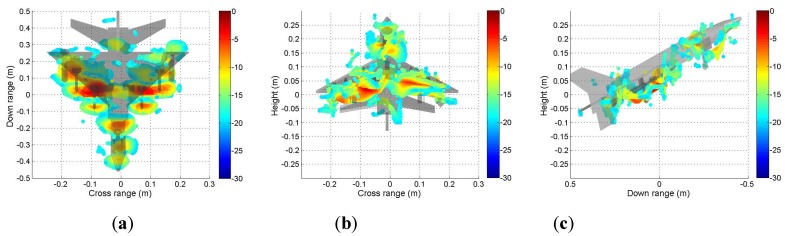
Tomography result in VV polarization. Three views of the airplane model are shown: (**a**) top view; (**b**) bottom view; (**c**) side view.

It can be seen from Figure 9 that the scattering mechanisms of the aircraft model are different in four polarizations. In the HH image, there are three strong scattering centers including two parts that are not distinguished (see Figure 9a). Compared with the HH image, more components can be distinguished from the VV image. The scattering intensity of the two cross-polarization images is low. Figure 10, Figure 11, Figure 12 and Figure 13 illustrate the 3D point cloud maps obtained from 24 2D images. The top views are similar to the 2D image, which proves that the 3D scattering intensity can be estimated by LSM. It can be seen from the bottom and side views that scattering centers with different heights are basically consistent with the aircraft model. In addition, the scattering intensity in front of the fuselage is higher than that of the fuselage tail due to the shielding of the supporting foam. By comparing the 3D point cloud maps in different polarizations, we can analyze its scattering mechanism.

**Figure 14:**
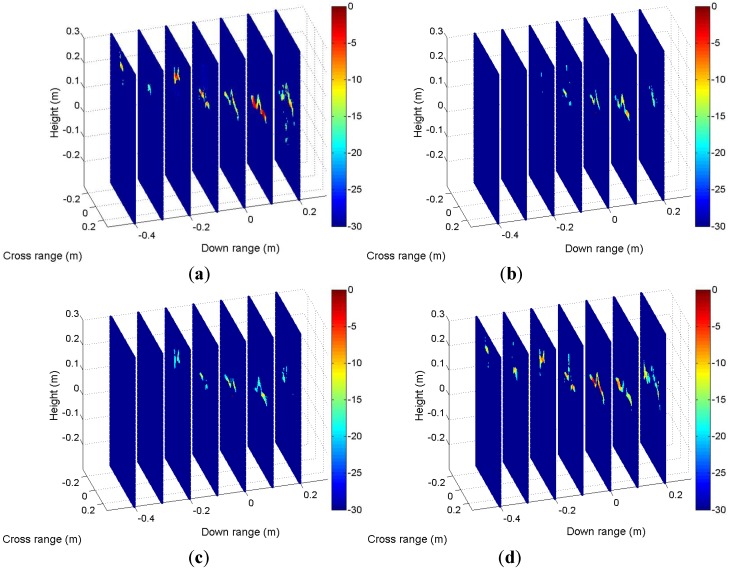
Tomographic image slices along the downrange: (**a**) HH, (**b**) HV, (**c**) VH, and (**d**) VV.

Figure 14 illustrates tomographic image slices along the down range for HH (Figure 10), HV (Figure 11), VH (Figure 12), and VV (Figure 13). It can be seen from the figures that scattering of the model shows obvious variety with heights. We summarize components of the model in Table 5, where M1 tail and M2 head cannot be distinguished because they have the same height, so do M2 tail and rear wheel.

## 6. Conclusions

This paper proposes a fully-polarimetric UMUSIC tomography algorithm to acquire high-resolution 3D radar imagery for a MIMO-SAR with a small number of baselines. In order to mitigate the effect of multi-looking on the range-azimuth resolution, we employ fully-polarimetric data and their conjugation to obtain the sample covariance matrix. Two algorithms including the fully-polarimetric DCS and the fully-polarimetric UMUSIC, are compared through numeric simulation of different point scatterers. Simulation results demonstrate that the fully-polarimetric UMUSIC outperforms the popular fully-polarimetric DCS in processing speed and estimation accuracy. Measurements for an aircraft model are conducted using an X-band experimental polarimetric MIMO-SAR which was upgraded from a previous system [7]. The resulting 3D images using six baselines demonstrate the usefulness of the algorithm for 3D imagery of complex radar targets.

## Figures and Tables

**Table 1:** Estimated results of two point scatterers with a spacing of 0.18 m.

			Cylinder (–0.09 m)	90° Rotated Dihedral Reflector (0.09 m)
Fully-polarimetric DCS	Height		−0.091 m	0.091 m
	Scattering intensity	HH	−1.8 dB	0 dB
		HV	−18.5 dB	−39.6 dB
		VH	−18.5 dB	−36.5 dB
		VV	−1.8 dB	0 dB
Fully-polarimetric UMUSIC	Height		−0.090 m	0.090 m
	Scattering intensity	HH	−1.8 dB	0 dB
		HV	−18.5 dB	−39.1 dB
		VH	−18.5 dB	−36.2 dB
		VV	−1.8 dB	0 dB

**Table 2:** Estimated results of two point scatterers with a spacing of 0.06 m.

			Cylinder (−0.06 m)	90° Rotated Dihedral Reflector (0 m)
Fully-polarimetric DCS	Height		−0.062 m	0.004 m
	Scattering intensity	HH	−3.0 dB	−1.6 dB
		HV	−17.6 dB	−29.5 dB
		VH	−17.6 dB	−37.5 dB
		VV	−3.2 dB	0 dB
Fully-polarimetric UMUSIC	Height		−0.060 m	0.004 m
	Scattering intensity	HH	−2.9 dB	−0.7 dB
		HV	−17.6 dB	−28.7 dB
		VH	−17.6 dB	−36.4 dB
		VV	−3.1 dB	0 dB

**Table 3:** Polarimetric scattering matrix (PSM) of four point scatterers

Parameters	Cylinder	67.5° Rotated Dihedral Reflector	90° Rotated Dihedral Reflector	Plate
HH	−1	$\sqrt{2}/2$	1	−1
HV	0	$\sqrt{2}/2$	0	0
VH	0	$\sqrt{2}/2$	0	0
VV	−1	−$\sqrt{2}/2$	−1	−1

**Table 4:** Estimated results of two point scatterers with a spacing of 0.09 m.

			Cylinder (−0.13 m)	67.5° Rotated Dihedral Reflector (−0.04 m)	90° Rotated Dihedral Reflector (0.05 m)	Plate (0.14 m)
Fully-polarimetric DCS	Height		−0.141 m	−0.030 m	0.041 m	0.141 m
	Scattering intensity	HH	−2.4 dB	−2.5 dB	−2.4 dB	−2.2 dB
		HV	−16.2 dB	−1.6 dB	−24.5 dB	−15.1 dB
		VH	−16.2 dB	−1.6 dB	−24.1 dB	−15.1 dB
		VV	−1.2 dB	−2.4 dB	−2.5 dB	0 dB
Fully-polarimetric UMUSIC	Height		−0.131 m	−0.039 m	0.057 m	0.140 m
	Scattering intensity	HH	−0.3 dB	0 dB	−1.7 dB	−0.5 dB
		HV	−14.6 dB	−2.4 dB	−36.8 dB	−15.1 dB
		VH	−14.6 dB	−2.4 dB	−37.2 dB	−15.1 dB
		VV	−0.3 dB	−4.0 dB	−3.0 dB	0 dB

**Table 5:** Components of the aircraft in tomographic image slices.

Down Range	−0.4 m	−0.3 m	−0.2 m	−0.1 m	0 m	0.1 m	0.2 m
Components	Aircraft head	Front wheel	Inlet	M1 head	M1 tail+ M2 head	M2 tail+ Rear wheel	M3 tail
HH	x	x	x	x	x	x	x
HV				x	x	x	
VH			x	x	x	x	
VV	x	x	x	x	x	x	x

## References

[1] Li J., Stoica P. (2007). MIMO radar with collocated antennas. IEEE Signal Process. Mag..

[2] Haimovich A.M., Blum R.S., Cimini L.J. (2008). MIMO radar with widely separated antennas. IEEE Signal Process. Mag..

[3] Ma Y., Miao C., Zhao Y.Y., Wu W. (2019). An MIMO Radar System Based on the Sparse-Array and Its Frequency Migration Calibration Method. Sensors.

[4] Narayanan R.M., Gebhardt E.T., Broderick S.P. (2017). Through-Wall Single and Multiple Target Imaging Using MIMO Radar. Electronics.

[5] Zhao D.Z., Jin T., Dai Y.P., Song Y.P., Su X.C. (2018). A Three-Dimensional Enhanced Imaging Method on Human Body for Ultra-Wideband Multiple-Input Multiple-Output Radar. Electronics.

[6] Zhou J., Zhu R., Jiang G., Zhao L., Cheng B. (2019). A Precise Wavenumber Domain Algorithm for Near Range Microwave Imaging by Cross MIMO Array. IEEE Trans. Microw. Theory Technol..

[7] Liu Y.Z., Xu X.J., Xu G.Y. (2018). MIMO radar calibration and imagery for near-field target scattering diagnosis. IEEE Trans. Aerosp. Electron. Syst..

[8] Shi Y., Zhu X., Bamler R. (2019). Nonlocal Compressive Sensing-Based SAR Tomography. IEEE Trans. Geosci. Remote Sens..

[9] Fornaro G., Pauciullo A., Reale D., Verde S. (2014). Multilook SAR Tomography for 3-D Reconstruction and Monitoring of Single Structures Applied to COSMO-SKYMED Data. IEEE J. Sel. Top. Appl. Earth Obs. Remote Sens..

[10] Aguilera E., Nannini M., Reigber A. (2012). Multisignal Compressed Sensing for Polarimetric SAR Tomography. IEEE Geosci. Remote Sens. Lett..

[11] Muhammad A.S., Tazio S., Irena H., Othmar F. (2019). A Case Study on the Correction of Atmospheric Phases for SAR Tomography in Mountainous Regions. IEEE Trans. Geosci. Remote Sens..

[12] Zhu X.X., Montazeri S., Gisinger C., Hanssen R.F., Bamler R. (2016). Geodetic SAR Tomography. IEEE Trans. Geosci. Remote Sens..

[13] Xing S.Q., Li Y.Z., Dai D.H., Wang X.S. (2013). Three-Dimensional Reconstruction of Man-MadeObjects Using Polarimetric Tomographic SAR. IEEE Trans. Geosci. Remote Sens..

[14] Liang L., Guo H.D., Li X.W. (2014). Three-Dimensional Structural Parameter Inversion of Buildings by Distributed Compressive Sensing-Based Polarimetric SAR Tomography Using a Small Number of Baselines. IEEE J. Sel. Top. Appl. Earth Obs. Remote Sens..

[15] Aghababaee H., Ferraioli G., Schirinzi G., Pascazio V. (2019). Regularization of SAR Tomography for 3-D Height Reconstruction in Urban Areas. IEEE J. Sel. Top. Appl. Earth Obs. Remote Sens..

[16] Li X.W., Liang L., Guo H., Huang Y. (2016). Compressive Sensing for Multibaseline Polarimetric SAR Tomography of Forested Areas. IEEE Trans. Geosci. Remote Sens..

[17] Nannini M., Scheiber R., Horn R., Moreira A. (2012). First 3-D Reconstructions of Targets Hidden Beneath Foliage by Means of Polarimetric SAR Tomography. IEEE Geosci. Remote Sens. Lett..

[18] Yang J.G., Jin T., Xiao C., Huang X.T. (2019). Compressed Sensing Radar Imaging: Fundamentals, Challenges, and Advances. Sensors.

[19] Fornaro G., Lombardini F., Serafino F. (2005). Three-Dimensional Multipass SAR Focusing Experiments with Long-Term Spaceborne Data. IEEE Trans. Geosci. Remote Sens..

[20] Pesavento M., Gershman A.B., Haardt M. (2000). Unitary Root-MUSIC with a Real-Valued Eigendecomposition: A Theoretical and Experimental Performance Study. IEEE Trans. Signal Process..

[21] Linebarger D.A., DeGroat R.D., Dowling E.M. (1994). Efficient direction-finding methods employing forward–backward averaging. IEEE Trans. Signal Process..

